# Genetically encoded biosensors for the circular plastics bioeconomy

**DOI:** 10.1016/j.mec.2024.e00255

**Published:** 2024-11-28

**Authors:** Micaela Chacón, Neil Dixon

**Affiliations:** Manchester Institute of Biotechnology (MIB), Department of Chemistry, University of Manchester, Manchester, M1 7DN, UK

**Keywords:** Genetic biosensors, Plastic, Degradation, Monomer synthesis, Biotechnology

## Abstract

Current plastic production and consumption routes are unsustainable due to impact upon climate change and pollution, and therefore reform across the entire value chain is required. Biotechnology offers solutions for production from renewable feedstocks, and to aid end of life recycling/upcycling of plastics. Biology sequence/design space is complex requiring high-throughput analytical methods to facilitate the iterative optimisation, design-build, test-learn (DBTL), cycle of Synthetic Biology. Furthermore, genetic regulatory tools can enable harmonisation between biotechnological demands and the physiological constraints of the selected production host. Genetically encoded biosensors offer a solution for both requirements to facilitate the circular plastic bioeconomy. In this review we present a summary of biosensors developed to date reported to be responsive to plastic precursors/monomers. In addition, we provide a summary of the demonstrated and prospective applications of these biosensors for the construction and deconstruction of plastics. Collectively, this review provides a valuable resource of biosensor tools and enabled applications to support the development of the circular plastics bioeconomy.

## Highlights

• The current linear plastic economic model is unsustainable.
• Biotechnology can offer circular plastic synthesis and recycling solutions.
• Genetically encoded biosensors are a powerful tool for quantifying chemical analytes.
• A number of plastic monomer-responsive biosensors have been developed.
• Biosensors have successfully guided biological plastic production and deconstruction.

## 1 Introduction

The invention of synthetic plastics in the 21st century revolutionised the modern world, transforming the landscape of medicine, transportation, and science and technology. They challenged and won against traditional materials in many areas of application, replacing steel, ceramic, wood and glass with lightweight and durable plastic polymers; lowering the fuel consumption of cars and aeroplanes, elongating the shelf-life of foods, and improving the energy efficiency of homes and buildings (Iacovidou et al., 2021). The entrenchment of plastics into everyday life is reflected by the generation of 350–400 million tonnes (Mt) of plastic worldwide per year, a figure that is expected to triple by 2050 (Geyer et al., 2017).

Despite the incredible advantages of these materials, they are at the forefront of two global issues: climate change and pollution. The plastics industry uses petrochemicals as a feedstock to generate the molecular building blocks for plastic polymers, consuming approximately 10% of the world's fossil fuel refinery output per year and generating about 4% of global greenhouse gas (GHG) emissions during the manufacturing process (Michaux, 2019; Tilsted et al., 2023). Further, the improper handling of end-of-life plastic has posed significant environmental challenges, with approximately 50% ending up in landfill and 22% evading management entirely – instead being leaked into the environment (OECD, 2022). None of the most abundantly produced plastics – including polyethylene (PE), polyethylene terephthalate (PET), polypropylene (PP), polystyrene (PS), polyamine (PA), polyurethane (PU), and polyvinylchloride (PVC) – are biodegradable, instead they persist and accumulate. This has resulted in near permanent contamination of the natural environment with plastic debris, severely impacting terrestrial and aquatic ecosystems alike (Geyer et al., 2017; Purohit et al., 2020).

While society's dependence on plastics cannot be undone, reform across their entire value chain is essential for the future without further planetary boundary encroachment (Villarrubia-Gómez et al., 2018). The future circular plastic economy will take many forms, two important facets of which will be (i) decoupling plastic production from petrochemical feedstocks and (ii) developing an effective end-of-life economy focused on reuse and recycling (Degli Esposti et al., 2021; World Economic Forum E. M. F. a. M. c., 2016). Here, biotechnology can offer solutions. Enzymatic depolymerisation has been gaining interest as a waste treatment strategy, with the last decade witnessing the discovery and characterisation of many plastic-degrading enzymes. A significant advantage to biocatalytic recycling is that the intrinsic value of the material is retained, offering a closed loop recycling technology (Tournier et al., 2023; Zhu et al., 2022). Further, whole-cell microbial biocatalysts have become an emerging tool for the production of plastic monomers. Here, biomass (lignocellulose, food waste, industrial byproduct) or captured CO_2_ can be used in the place of petrochemicals as a feedstock for the production of renewable plastics (Choi et al., 2023; Degli Esposti et al., 2021).

Biological production and degradation of plastics, however, is still in its nascence. Bringing this arm of biotechnology closer to replacing the incumbent production/destruction technologies will involve novel strain/enzyme discovery and engineering. Such work often involves the extensive screening of libraries, a challenging endeavour that can be aided by the use of genetically encoded biosensors (Yu et al., 2023). Biosensors are genetic regulatory systems used by cells to perceive changes in environmental conditions or metabolite levels. The bio-sensing of a molecular signal (input) is then used to proportionally actuate a response (output), such as the transcription or translation of relevant genes/proteins (Alvarez-Gonzalez and Dixon, 2019; Chaisupa and Wright, 2024). Synthetic biology has harnessed genetically encoded biosensors to detect and quantifiably measure analyte levels, pairing this input with a desired output – such as the expression of a fluorescent protein or genetic pathway. The development of whole cell biosensors has streamlined the screening of microbial and enzyme libraries, shortening detection time compared to traditional methods such as chromatography and mass spectrometry, and permitting larger sampling sizes (Kaczmarek and Prather, 2021). Further, the high temporal resolution of a biosensor to an analyte has been harnessed for the self-regulation of microbial metabolic pathways, permitting a dynamic balance between productivity and cell fitness in production strains.

In the context of the plastic economy, genetically encoded biosensors are a tool that can be leveraged to accelerate the discovery and optimisation of plastic-acting enzymes for biological plastic recycling and renewable plastic production by microbial hosts. In this review we will discuss the availability of plastic monomer responsive genetically encoded biosensors, the work done towards engineering a number of these biosensors for desired purpose, and their application in biological plastic construction and deconstruction.

## 2 Biosensors

Genetically encoded biosensors permit the conversion of an input signal, most commonly a small molecule metabolite, into a gene expression output, such as a reporter or functional gene. As such, biosensors permit sensing, regulation and control in response to effector molecules and therefore have applications, in diagnostics, environmental monitoring, bio-process control, and as an enabling tool for discovery and optimisation of enzymatic and cellular processes (Bayer et al., 2023; Chiang and Hasty, 2023; Mitchler et al., 2021; Otero-Muras and Carbonell, 2021; Yi et al., 2021). Genetically encoded biosensors can broadly be categorised based on the type of receptor molecule used for ligand-binding, namely nucleic acid-based or protein-based biosensors. Regarding the latter, such protein-based biosensors can permit ligand sensing via an array of different molecular mechanisms, either by harnessing the sensing and regulatory components found in nature, or by developing engineered chimeric or wholly artificial sensing components. Examples of protein-based biosensors include allosteric transcription factor based sensors, membrane protein based sensors (including mechanosensitive channel based sensors), immuno-based sensors, and enzyme based sensors (Chaisupa and Wright, 2024).

Allosteric transcription factors (aTF) have most commonly been employed due their relatively simple architecture, direct connection between the input and output components, and their ability to sense intracellular metabolites. Such aTF-based biosensors are comprised of a genetic circuit whereby upon effector binding the expression of an output gene is activated or de-activated. In its simplest format, a biosensor might comprise a repressor based aTF that binds to its corresponding promoter-operator sequence in the absence of the effector molecule, and therefore blocks access to the promoter by the host transcription machinery, RNA polymerase (RNAP) (Browning and Busby, 2016). Upon effector binding, the aTF loses its affinity for the operator sequence, permitting de-repression and access to the promoter by RNAP.

**Fig. 1:**
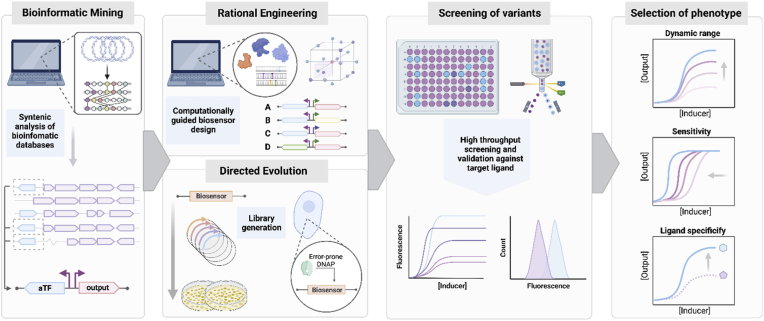
Steps towards the development and optimisation of a transcriptional biosensor. Bioinformatics can be used to identify candidate aTF-promoter pairs. Rational engineering or directed evolution can be employed to create a library of biosensor variants which can then be evaluated using high throughput techniques such as well plate screening, FACS (Fluorescence-Activated Cell Sorting), and agar plate-based screening. From the library analysis, biosensors with desired characteristics (improved dynamic range, altered sensitivity, change in ligand specificity, etc) can be selected for.

The first step to identifying the components required to construct a transcriptional biosensor is commonly a bioinformatic step, whereby syntenic analysis is used by querying bioinformatic databases with genes associated with the metabolism or transport of the effector of interest in order to identify candidate aTF-promoter pairs associated with the same genomic loci (Fig. 1). The DNA encoding such pairs can then be transferred directly into the required host or it may require host-context optimisation. This optimisation can entail modification of the regulatory elements (promoter and RBS elements), codon optimisation of the gene coding regions, or indeed development of chimeric promoter-operators by splicing defined operator sequences into define host promoter regions (Machado and Dixon, 2016; Rondon et al., 2019).

As well, beyond these host-context optimisations, further engineering may be required to enhance the performance metrics of the biosensor, such as improving the dynamic range and sensitivity of the response, or decreasing the synthetic burden imposed upon the host by the biosensor circuit (Ang et al., 2013; Berepiki et al., 2020; Ding et al., 2020; Mitchler et al., 2021). In order to permit these changes in biosensor performance, the encoded genetic regulatory elements can be targeted to attenuate the stoichiometry of the biosensor components. Other more significant changes may involve the modification of the DNA-binding domains (DBD) of the aTF, or similarly, modification of the corresponding promoter operator sequence that binds to the DBD (Alvarez Gonzalez et al., 2024; Rondon et al., 2019). A number of approaches can be employed to sample the design space of these biosensor circuits for the purposes of engineering its function, including i) mechanistically driven approaches, ii) the use of fractional factorial sampling of structured genetic libraries, or iii) stochastic introduction of point mutations within regulatory components (De Paepe et al., 2017; Javanpour and Liu, 2021; Koch et al., 2019; Rondon et al., 2019). Across these approaches different levels of prior knowledge are required, from one extreme requiring in depth biochemical and structural knowledge of the regulatory mechanism to the other extreme employing a blind shotgun approach to protein/genetic circuits engineering (Fig. 1). Irrespective of the approach employed to generate biosensor circuit variants, common approaches are employed to screen for perturbation of the performance of resultant biosensors. Fluorescent reporter proteins are the mostly commonly used output due their ease of use for both clonal library screening, via multi-well screening with a plate reader, or polyclonal library sorting, via FACS-based approaches (Fig. 1).

## 3 Genetically encoded biosensors for plastics

Numerous genetically encoded biosensors have been developed to date that are responsive to common monomers used in the construction of plastics, including diacids, diamines, diols, hydroxyacids, lactones, lactams, amino acids, etc (Table 1). Here we discuss those biosensors, the genetic components used in their construction, and briefly summarise work performed to integrate them into the host strain genetic regulatory network and/or improve their performance for specific applications.

**Table 1:**
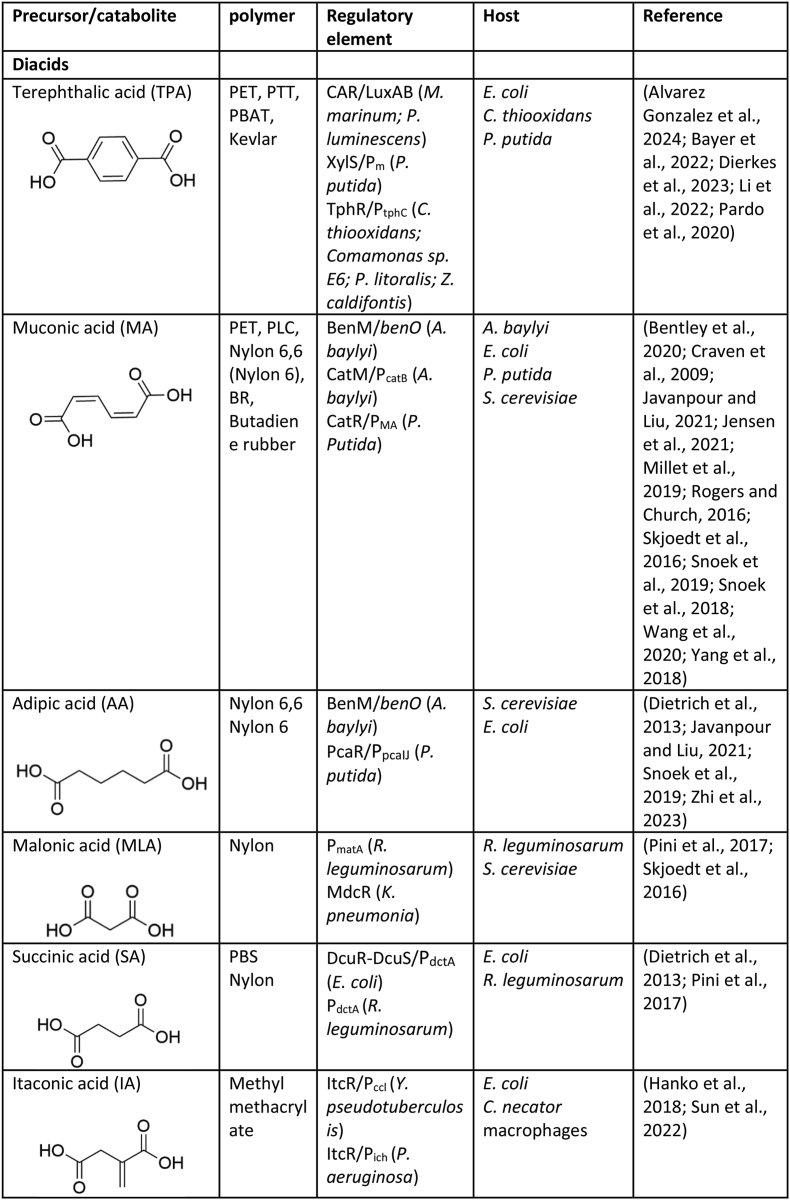

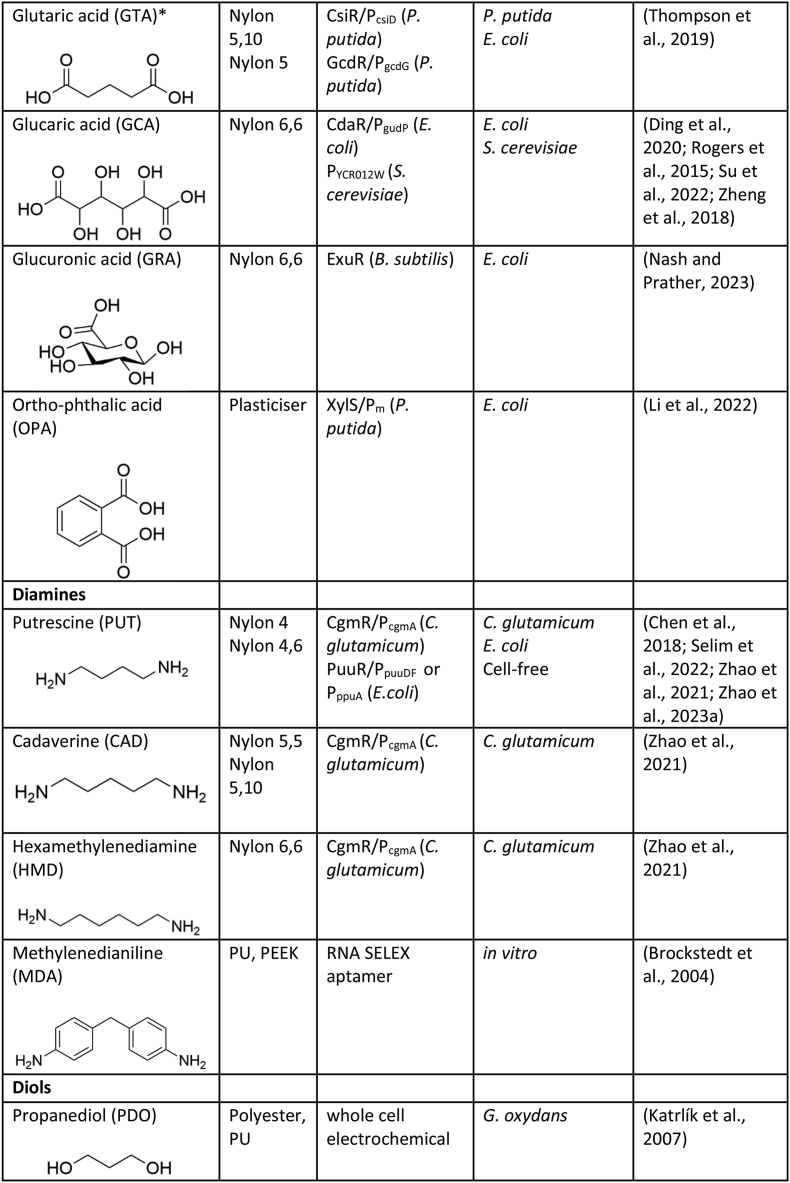

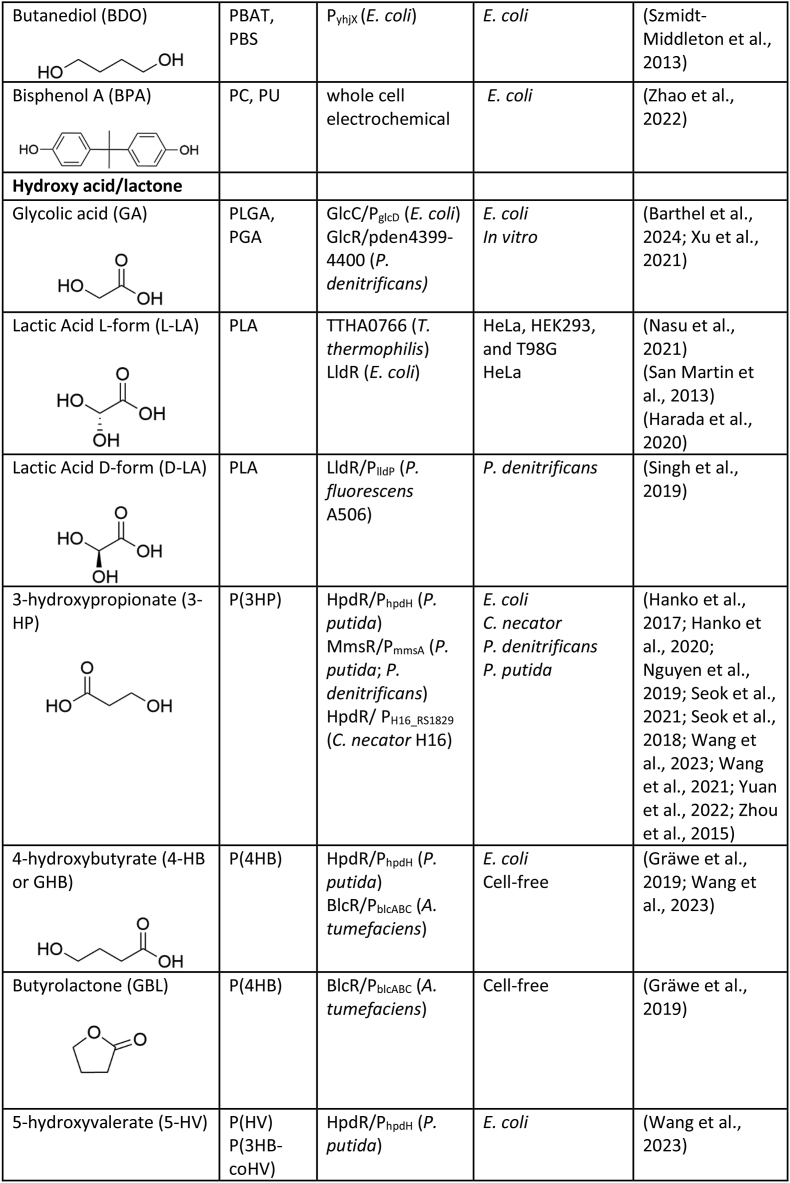

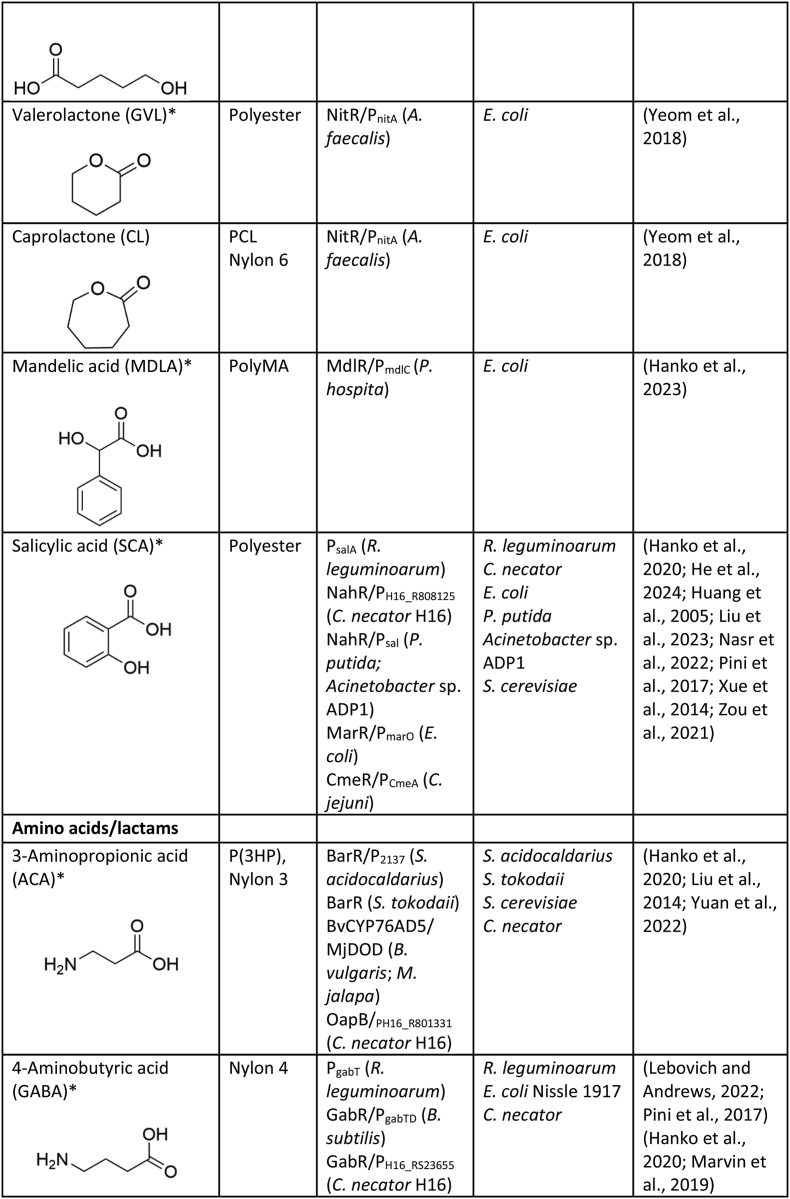

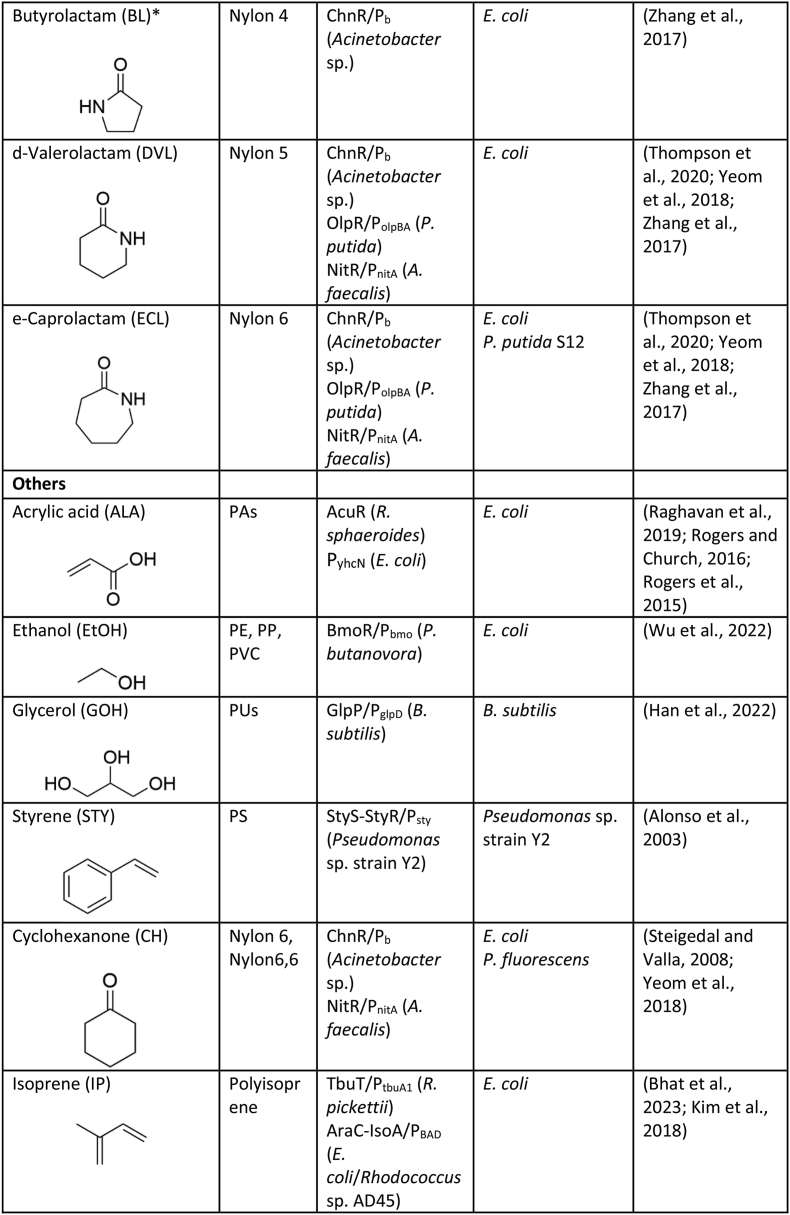
Genetically encoded biosensors for common plastic monomers.

∗These compounds are not currently as widely used or commercially relevant in large-scale plastic production, though they have potential applications in niche or emerging technologies.

### 3.1 Diacids

Diacids are commonly used monomers for direct incorporation into block co-polymers e.g., ester- and amide-based plastics, or as precursors for the subsequent conversion into monomers e.g., butadiene and methyl acrylate (Lansing et al., 2017; Mori et al., 2021).

#### 3.1.1 Muconic acid

To date the most highly reported diacid-responsive biosensor is for the C6 diacid, muconic acid (MA). MA is a precursor to adipic acid, terephthalic acid, and 6-hydroxy-caproic acid, used in the production of nylon 6,6, and polyethylene terephthalate (PET), (along with a variety of other ester and amide-based plastics), and butadiene rubber (Mori et al., 2021). MA biosensors have been developed utilising a number of different MA-responsive allosteric transcription factors, including BenM (Craven et al., 2009; Rogers and Church, 2016; Millet et al., 2019) and CatM (Bentley et al., 2020), which are both LysR-type transcriptional regulators (LTTR) from Acinetobacter sp. strain ADP1 (Tropel and van der Meer, 2004). As well, CatR, another MA-responsive LTTR from *Pseudomonas putida* KT2440 (Yang et al., 2018), has also been employed for MA biosensor construction. Beyond exploitation of these biosensors in bacteria, the MA-responsive BenM biosensor has also been transplanted into *Saccharomyces cerevisiae*. Here (Skjoedt et al., 2016), placed the operator site of the BenM DNA-binding site (BenO) into a eukaryote promoter (CYC1p) to create a functional hybrid MA responsive biosensor in *S. cerevisiae*. To further improve the dynamic range of the MA biosensor, a library of BenM variants was generated using PCR mutagenesis, with one variant containing three point mutations in the effector binding domain (EBD) identified as having double the dynamic range compared to the wild-type BenM. This *S. cerevisiae* transplanted BenM biosensor was later employed in both adaptive laboratory evolution (Snoek et al., 2018) and directed evolution (Jensen et al., 2021) for strain optimisation to improve MA titre, and was also subjected to protein engineering to change the effector specificity towards the saturated C6 diacid, adipic acid (AA) (Snoek et al., 2019). A number of the reported MA biosensors display significant off-target activity, with activation observed with adipic acid (AA) (Javanpour and Liu, 2021). To overcome this undesired off-target activity and expand the dynamic range of the BenM-derived MA biosensor further, Javanpour and Liu (2021) employed a continuous *in vivo* hypermutation system termed OrthoRep. Firstly creating highly diverse BenM variants utilising an *in vivo* orthogonal error prone DNA polymerase step before transferring these BenM variants into a second *S. cerevisiae* reporter strain for FACS-based enrichment, this approach afforded a MA biosensor, named CCM-4, with a dynamic range of ∼180-fold and no off-target activation with AA (Javanpour and Liu, 2021).

#### 3.1.2 Adipic acid

Biosensors responsive towards another C6 diacid, the reduced adipic (AA), a precursor for nylon 6 and nylon 6,6, have also been reported. A number of the C6 diacid responsive biosensors display limited selectivity, responding to both MA and AA (Javanpour and Liu, 2021). To improve AA selectivity, a targeted diversification of the EBD domain of the BenM regulator followed by FACS toggled selection was employed. From this, a BenM variant named TISNO-120 containing four point mutations demonstrated increased sensitivity towards AA and reduced sensitivity towards MA (Snoek et al., 2019). However, TISNO-120 is reported to display only a 5-fold difference in saturation response between the cognate AA and the off-target MA ligand (Javanpour and Liu, 2021). More recently, efforts to improve the BenM biosensor response to AA and reduce off-target activation have employed OrthoRep, a continuous *in vivo* hypermutation system. This approach afforded a biosensor variant, named AA-5, with a dynamic range of ∼180-fold, and a 24-fold difference in saturation response between the cognate AA and the off-target MA ligand (Javanpour and Liu, 2021). Finally, the beta-ketoadipate responsive transcription factor, PcaR, has been employed as an AA biosensor affording conditional antibiotic resistance, using the *tetA* reporter gene, in response to AA and other saturated C4-C7 diacids (Dietrich et al., 2013).

#### 3.1.3 Terephthalic acid

The aromatic diacid, terephthalic acid (TPA), is a widely utilised monomer within a number of different polyester and aramide plastics e.g., polyethylene terephthalate (PET) and Kevlar, respectively. Biosensors for TPA have been developed based on the IclR-type TPA-responsive regulator, TphR derived from the *tph* operon*,* encoding the genes *tphCA*_*2*_*A*_*3*_*BA1* for TPA transport and catabolism (Gautom et al., 2021; Kasai et al., 2010). Firstly, a TPA biosensor based on TphR and its responsive promoter from *Comamonas* sp. was developed in an *Acinetobacter baylyi* host, with iterative optimisation of the regulatory elements (RBS and hex boxes) upstream from the *eGFP* reporter affording the best biosensor, pTPA3, with a dynamic range of ∼6-fold in response to TPA (Pardo et al., 2020). Another biosensor was developed in the native *tph* operon containing host *Comamonas thiooxidans S2*3 by placing the *eGFP* reporter downstream from the *tphC* promoter on a plasmid (TphR remained genomically encoded). This afforded a TPA responsive biosensor with a dynamic range of >25-fold and ∼4-fold in the wild-type and *ΔtphA2A3BA1* background, respectively (Dierkes et al., 2023). Genetic circuit engineering of a TphR-based biosensor has also been reported to enable attenuation of various dose-response curve metrics for different screening applications (Alvarez Gonzalez et al., 2024). Here a series of TphR transcription factors and their responsive promoters were identified through bioinformatic prospecting, and subsequently assembled for biosensor screening in a *Pseudomonas putida* host. The best TPA biosensor, based on genetic parts mined from *Z. caldifontis,* was then refactored and subjected to a design of experiment workflow established to improve dynamic range and attenuate the slope of the dose-response curve. This afforded TPA biosensors with dynamic ranges of >12-fold and variously tuned dose response curve parameters, including slope steepness from digital to analogue (n_H_ 0.2 to 2.2). A TPA-responsive biosensor has also been developed utilising XylS, a promiscuous transcription factor from *Pseudomonas putida* subsequently engineered using site directed saturation mutagenesis to respond to number phthalic acids, including TPA, ortho-phthalic acid (OPA), and, to a smaller extent, isophthalic (IPA) (Li et al., 2022). Isophthalic is a monomer for the aramide NOMEX, whilst ortho-phthalate is a precursor for the commonly used plasticiser diethylhexyl phthalate (DEHP). Finally, a semi-quantitative enzyme coupled biosensor for TPA has been reported, whereby TPA is initially reduced by carboxylic acid reductase to the corresponding aldehydes, which are then re-oxidised by LuxAB monooxygenase to the carboxylic acids, emitting a bioluminescent signal with a dynamic range 17-fold in response to TPA.

#### 3.1.4 C3 – C6 diacids

The C3 diacid, malonic acid (MLA), is a monomer for short-chain nylons, and a number of biosensors have been developed that respond to it (Pini et al., 2017; Skjoedt et al., 2016). The transcriptional regulator of the MLA catabolic pathway in *Klebsiella pneumonia*, MdcR (Peng et al., 1999), was used to establish an MLA biosensor in *Saccharomyces cerevisiae,* affording a 2-fold dynamic range (Skjoedt et al., 2016). In addition an MLA biosensor using the GntR-family transcriptional regulator, MatR, in a *lux-*based reporter system in *Rhizobium leguminosarum* was reported to afford a dynamic range of 540-fold (Pini et al., 2017).

The C4 diacid, succinic acid (SA), is a monomer for nylon-4,4 and poly (butylene succinate) (PBS). SA responsive biosensors have been constructed by coupling either the beta-ketoadipate responsive transcription factor, PcaR, from *P. putida* or the DcuS/DcuR two-component system from *E. coli*, and their natively regulated promoters, to an antibiotic an resistance gene (*tetA*) in order to afford conditional growth on tetracycline in the presence of succinate (Dietrich et al., 2013). In addition, using the regulator and promoter region upstream from the dicarboxylate transporter gene, *dctA,* from *Rhizobium leguminosarum* coupled to *lux-*based reporter resulted in a biosensor with a dynamic range of 36-fold in the presence of SA (Pini et al., 2017).

The C5 diacid, icatonic acid (IA), is a precursor for methyl methacrylate (MM). An IA biosensor has been developed based on the LTTR-type IA-responsive regulator, ItcR, from *Yersinia pseudotuberculosis* in both *E. coli* and *Cupriavidus necator* hosts, affording dynamic ranges of 215- and 105-fold, respectively in the presence of IA (Hanko et al., 2018). More recently a protein-based biosensor has been developed for the detection of IA in the mitochondria and cytosol of living macrophage. This biosensor employs the effector binding domain of the ItcR along with a circularly permuted green fluorescent protein (cpGFP) (Sun et al., 2022). Although the dynamic range of the biosensor is lower than those based on transcriptional DNA-binding/regulation, this is an interesting approach for host independent biosensing. Another C5 diacid, Glutaric acid (GTA), is a precursor for Nylon 5,10. Biosensors for GTA have been reported based on the *P. putida* L-lysine catabolism associated GntR-type regulator, CsiR, and the LTTR-type regulator, GcdR. Expression of these biosensors in an *E. coli* host resulted in a 55-fold dynamic range in the presence of GTA by the GcdR biosensor (Thompson et al., 2019).

The C6 diacid, glucaric acid (GCA), is a precursor for other monomers used in plastic synthesis, including Nylon-6,6. A biosensor for GCA has been developed based on the CdaR regulator from *E. coli* (Rogers et al., 2015), and exploited to monitor endogenous GCA production both in *E. coli* (Rogers and Church, 2016) and in *S. cerevisiae* via a tandem co-culture approach (Zheng et al., 2018). The *E. coli*-based GCA biosensor has been further improved by screening a large RBS library to attenuate the protein (translation initiation) levels and stoichiometry of the regulator and reporter in order to reduce metabolic burden associated with the biosensor. This ultimately extended performance from a reported starting point of ∼10-fold to >200-fold dynamic range (Ding et al., 2020). More recently, a GCA responsive promoter has been characterised in *S. cerevisiae.* Although the study didn't confirm the identity of the corresponding transcriptional regulator, a series knock-out strains indicated that the regulators Ash1p and Cbf1p might be mediating the GCA biosensing (Su et al., 2022).

Finally, the C6 diacid, glucuronic acid (GRA), is a precursor for GCA and hence Nylon 6,6. A biosensor for GRA has been developed based on the transcriptional regulator of the galacturonic acid transport and catabolism genes in *Bacillus subtilis,* ExuR, which has been reported to respond to both galacturonic and GRA (Mekjian et al., 1999). This biosensor was constructed using a hybrid promoter upstream of *sfGFP* whereby the ExuR DNA binding sites were inserted into a proD promoter. Expression of the GRA biosensor in a *uxaC* knockout strain of *E. coli* – to prevent isomerisation of GRA to fructuronate – resulted in a dynamic range of ∼20-fold and an operational range of 0.01–1 g/L GRA (Nash and Prather, 2023; Ni et al., 2022).

### 3.2 Diamines

Diamines are commonly used as monomers for direct incorporation into amide-based and carbamate-containing plastics e.g. nylons, aramides, and polyurethanes.

#### 3.2.1 C4-C6 diamines

The C4 diamine, putrescine (PUT), is a monomer for nylon-4,4 and nylon 4,6. Biosensors for PUT have been developed using the *E. coli* transcriptional repressor, PuuR, which natively regulates the *puu* genes involved in PUT metabolism. An engineered PUT biosensor for *E. coli* was constructed using a hybrid promoter upstream of a *gfp* reporter whereby the PuuR DNA-binding site (PuuO) was placed into a TacR2 promoter. The resulting biosensor afforded the selective detection of the C4 diamine with a dynamic range of ∼7-fold (Chen et al., 2018). Further, the PuuR biosensor has been established as a cell-free PUT biosensor for use as a portable technology, with application in the testing of meat stability and quality assessment (Selim et al., 2022). The PuuR biosensor has since been established in a *Corynebacterium glutamicum* diamine production host (Zhao et al., 2023a). Here it was found that PuuR is responsive to PUT and partially to 1,3-propylenediamine (used as a chain extender in polyurethanes), affording dynamic ranges of 9- and 3-fold, respectively. In parallel another PUT biosensor was developed based on the TetR-family transcriptional repressor, CgmR, from *Corynebacterium glutamicum* (Nguyen et al., 2015). Through systematic protein and genetic circuit engineering approaches, the performance of the CgmR biosensor was improved and the resulting biosensor variant, named pSenPut_I152T_, had a dynamic range of 60-fold in response to PUT (Zhao et al., 2021).

Further, it has been identified that the CgmR regulator from *Corynebacterium glutamicum* responds to the C5 diamine, cadaverine (CAD), and the C6 diamine, hexamethylenediamine (HMD), in addition to PUT. CAD is a monomer for nylon-5 and nylon 5,10 while HMD is a monomer for nylon 6,6. The engineered pSenPut_I152T_ biosensor developed by (Zhao et al., 2021) afforded a ∼90-fold and ∼60-fold dynamic range in response to CAD and a HMD, respectively.

#### 3.2.2 Methylenedianiline

The aromatic diamine, methylenedianiline (MDA), is a monomer for polyurethane (PU) and a precursor for polyetheretherketone (PEEK). Although no biosensor for MDA has been reported, an RNA aptamer has been discovered using a SELEX screening approach, opening up the possibility for a riboswitch-based biosensor for MDA detection (Brockstedt et al., 2004).

### 3.3 Diols

Diols are important monomers for a range of ester and carbamate-based plastics including PET, polybutylene succinate (PBS) and polyurethanes. To date, only one biosensor for 1,4 butanediol (BDO) has been reported (Szmidt-Middleton et al., 2013) based on the BDO responsive promoter upstream of *yhjX* (which encodes a putative efflux pump) in *E. coli*. Although the responsive regulator has not been identified, the P_yhjX_::GFP construct afforded >10-fold dynamic range in response to 5% BDO in its native host.

A whole cell amperometric biosensor has been reported for the detection of 1,3-propanediol (PDO), which is used for the production of polyesters and polyurethane. The PDO biosensor is based on *Gluconobacter oxydans* cells and was able to selectively detect the production of PDO from glycerol (Katrlík et al., 2007). Another whole cell biosensor with an electrochemical read-out was reported for Bisphenol A (BPA), which is used for in the production polyurethane and polycarbonate plastics. The BPA biosensor is based on the use of engineered *E. coli* cells surface displaying a recombinant tyrosinase, and displayed a detection limit of 10pM (Zhao et al., 2022).

### 3.4 Hydroxy acids and lactones

Hydroxy acids, and the corresponding cyclised lactones, are monomers for a wide variety of ester-based synthetic plastics, nylons and biopolymers including polylactic acid (PLA), and polyhydroxyalkanoate (PHA).

#### 3.4.1 Glycolic acid

The C2 hydroxy acid, glycolic acid (GA), is monomer used in the production of both biodegradable polyglycolic acid (PGA) and poly lactic-co-glycolic acid (PLGA). A GA biosensor based on the GntR-type transcriptional activator, GlcC, from *E. coli* has been developed. By optimizing the expression level of the GlcC regulator, a biosensor with a reported dynamic range of 80-fold and an operational range between 0.1 and 200 mM GA has was achieved (Xu et al., 2021). Recently, an *in vitro* transcription based biosensor for the detection of GA was developed based on the GntR-type transcriptional repressor, GlcR, and the intergenic region to which it binds (pden4399-4400), from *Paracoccus denitrificans*. The GA responsive biosensor, controlling expression of the 3WJdB RNA aptamer which results in a green-fluorescent signal by interacting with and stabilising the DFHBI -1T dye, was used to detect GA production from CO_2_ by the in *vitro* CETCH cycle. This GA biosensor was found to have a dynamic range of ∼10-fold and operational range of 16 μM- 8 mM (Barthel et al., 2024).

#### 3.4.2 Lactic acid

The C3 hydroxy acid, lactic acid (LA), is a monomer for polylactic acid (PLA), the D and L stereoisomers of LA can be polymerised to make PDLA, PLLA or a racemic mixture called PDLLA. A biosensor selective for D-lactic acid has been reported based on GntR-family protein, D-LldR from *Pseudomonas fluorescens.* When established in a *Pseudomonas denitrificans* host this biosensor afforded a dynamic range of ∼6-fold in the presence of D-LA (Singh et al., 2019). Another LA detection approach has involved the development of an extracellular sensor using a circularly permuted green fluorescent protein (cpGFP) and the L-lactate periplasmic binding protein, TTHA0766, from *Thermus thermophilis*. The optimised biosensor, eLACCO1.1, afford >5-fold dynamic range in the presence of L-LA, although the output signal was highly dependent upon environment factors, and also showed partial response to D-LA (Nasu et al., 2021). Further, a single fluorescent protein-based biosensor has been reported for LA based on the development of a chimer of GFP and the effector binding domain of lactate dehydrogenase transcriptional regulator, LldR, from *E. coli* (Harada et al., 2020). The resulting biosensor, named Green Lindoblum, afforded a dynamic range of ∼5-fold in the presence of LA. Finally, a genetically encoded FRET-based biosensor has been reported, employing the lactate dehydrogenase transcriptional regulator, LldR, and N and C terminal fluorophore-quencher pairs, mTFP-Venus, the optimised biosensor was able to detect LA between 1 μM and 10 mM (San Martin et al., 2013).

#### 3.4.3 C3-5 hydroxy acids

Another C3 hydroxy acid, 3-hydroxypropionic acid (3-HP), is a monomer for the short chain polyhydroxyalkanoate (PHA) bioplastic, poly (3-hydroxypropionate) P (3HP). PHAs possess similar material characteristics to conventional plastics and are biodegradable, making them attractive bioplastics (Chaisupa and Wright, 2024). An enzyme-coupled biosensor for 3-HP detection was developed by pairing the 2-methylcitrate (2-MC) responsive transcriptional regulator, PrpR, from *E. coli* with the biotransformation of 3-HP into 2-MC via *pcs* (propionyl-CoA synthase) and *prpC* (2-methylcitrate synthase) in an *E. coli* host. The resulting enzyme cascade-biosensor pair afforded a 3.5-fold dynamic range in response to 3-HP (Rogers and Church, 2016). A second enzyme-coupled biosensor was also developed by pairing the acrylate-responsive regulator, AcuR, from *Rhodobacter sphaeroides* with the biotransformation of 3-HP to acrylate via a truncated *pcs* and *ach* (acrylyl-CoA hydrolase). This coupled biosensor afforded a 90-fold dynamic range in response to 3-HP addition (Rogers and Church, 2016). Through transcriptional analysis of the putative 3-HP catabolic genes in *P. dentrificans*, two LysR-type aTFs (named C3-LysR and C4-LysR) where implicated as being regulated by this effector (Zhou et al., 2015). The C4-LysR regulator from *P. denitrificans,* MmsR, was subsequently employed to develop a biosensor-selection device, pCDF-3HPselector, in *E. coli,* whereby either *tetA* or *eGFP* expression was attenuated in the presence of 3-HP, affording a dynamic range >5-fold (Seok et al., 2018). Nguyen et al. (2019) also developed a 3-HP responsive biosensor based on the MmsR transcriptional activator from *P. dentrificans*. Through engineering efforts targeting expression of the regulator and the sequence of the corresponding P_mmsA_ promoter, a biosensor with ∼100-fold dynamic range in response to 3-HP was developed. Similarly, two 3-HP biosensors were developed by identifying 3-HP up-regulated catabolic genes and the corresponding promoter-regulator pairs from *P. putida*, MmsR/P_mmsA_ and HpdR/P_hpdH_. These LTTR-based biosensors were reported to afford a dynamic range of ∼12- and 23-fold, respectively, when assessed in their native host, *P. putida,* and ∼52- and >500-fold, respectively, when assessed in *C. necator* (Hanko et al., 2017). The HpdR/P_hpdH_ components from *P. putida* have also been used to construct a 3-HP biosensor in *E. coli,* whereby 3-HP is employed as a downstream proxy for the production target, beta-alanine (Yuan et al., 2022). Further, another biosensor based on the HpdR/P_hpdH_ parts from *P. putida* was engineered by deleting a palindromic sequence in the corresponding P_hpdH_ promoter. The resulting promiscuous biosensor displayed dynamic ranges of ∼14-fold, ∼14-fold and ∼20-fold in response to 3-HP, 4-hydroxybutyrate and 5-hydroxyvalerate, respectively, when expressed in *E. coli* (Wang et al., 2021, 2023).

The C4 hydroxy acid, 4-hydroxybutyrate (4-HB), also known as gamma-hydroxybutyric acid or GHB, is a precursor for the PHA bioplastic, poly (4-hydroxybutyrate) P (4HB). Likewise, the C5 hydroxy acid, 5-hydroxyvalerate (5-HV), is a precursor for poly (5-hydroxyvalerate) P (5HV) and poly (3-hydroxybutryate-*co*-5-hydroxyvalerate) P (3HB-*co*-5HV). A cell free biosensor was developed to detect illicit use of 4-HB, based on the BlcR regulator from *Agrobacterium tumefaciens,* affording a dynamic range of ∼8-fold in the presence of 4-HB (Gräwe et al., 2019). In addition to detecting linear 4-HB, the cell free BlcR-based biosensor was also reported to detect the cyclic lactone γ-butyrolactone (GBL), affording a dynamic range of ∼8-fold in the presence of GBL (Gräwe et al., 2019).

#### 3.4.4 Valerolactone and caprolactone

Gamma-valerolactone (GVL) is a precursor to polyesters, while caprolactone (CL) is a precursor to various plastics including polycaprolactone (PCL) and nylon 6. A biosensor based on the transcriptional regulator, NitR, from *Alcaligenes faecalis* has been developed in *E. coli*, which afford a dynamic range of >10-fold in the presence of either GVL and CL (Yeom et al., 2018).

#### 3.4.5 Mandelic acid

The aromatic hydroxy acid, mandelic acid (MDLA) is a precursor to the biopolymer (poly)mandelic acid (PMA). A biosensor for S-mandelic acid has been developed based on the LysR-type transcriptional regulator, MdlR, from *Paraburkholderia hospita* in the heterologous host, *E. coli* (Hanko et al., 2023). The MdlR-based biosensor afforded a dynamic range of >15-fold and ∼8-fold in the presence of MDA and the closely related compound, phenylglyoxylate, respectively.

#### 3.4.6 Salicylic acid

Salicylic acid (SCA) has been incorporated into a variety of polyester-based bioplastics to improve their hydrolytic degradability (Kim et al., 2021). A biosensor for SCA has been developed using the regulator and promoter region upstream from the salicylic acid export system, SalA, from *Rhizobium leguminosarum* coupled to *lux-*based reporter biosensor afforded dynamic range of >1000-fold in the presence of SCA (Pini et al., 2017). Further, an SCA biosensor based on the LysR type transcriptional activator, NahR, from *C. necator*. When expressed in the native host this biosensor demonstrated a dynamic range of ∼650-fold in response to SCA, as well as the ability to detect nM concentrations of this effector. It was additionally found to be active in the heterologous hosts, *E. coli* and *P. putida* (Hanko et al., 2020). Another biosensor based on NahR from *P. putida* was also developed for *E. coli*, here a dynamic range of ∼90-fold in response to SCA was achieved (Xue et al., 2014). Several groups have undertaken engineering efforts to improve the NahR based biosensor in *E. coli*, using multiple strategies to target expression of the regulator and output proteins in response to SCA (He et al., 2024; Liu et al., 2023). *Acinetobacter* sp. ADP1 has also been converted into a whole-cell SCA detector by integrating either GFP or luxCDABE into the genome within native operon regulated by NahR (Huang et al., 2005). Recently, Zou et al. (2021) developed and SCA biosensor in *E. coli* by exploiting the native antibiotic resistance regulator, MarR, which responds to SCA. Here, the operator sites of the corresponding P_marO_ promoter were inserted into the constitutive pL promoter, resulting in ∼4.5-fold dynamic range in response to SCA. As well, recently the broad spectrum Tet-family transcriptional repressor, CmeR, from *Campylobacter jejuni* has been converted into a SCA biosensor in *E. coli* and *S. cerevisiae*, demonstrating a >5-fold and ∼4.3-fold in response to this effector, respectively (Nasr et al., 2022).

### 3.5 Amino acids and lactams

Amino acids, and the corresponding cyclised lactams, are monomers for a wide variety of amide-based plastics such as nylons.

#### 3.5.1 3-Aminopropionic acid

3-Aminopropionic acid (ACA), or β-alanine, is a precursor for the bioplastic P (3HP) via the 3-HP, and as a monomer for Nylon 3. An ACA biosensor was reported in *Sulfolobus acidocaldarius* based on the endogenous Lrp-like regulator BarR (Liu et al., 2014). Replacement of the native functional genes with *lacZ* reporter gene afforded a biosensor with a dynamic ∼2.5-fold in the presence of ACA. Another ACA biosensor was reported in *C. necator* based on the endogenous MocR family regulator, OapR (Hanko et al., 2020). In the native host, this biosensor demonstrated an ∼ 8-fold dynamic range in response to ACA. Further, by swapping the native P_OapR_ promoter and RBS regulating expression of the aTF to a constitutive *E. coli* promoter, the biosensor was found to be functional in this heterologous host (Hanko et al., 2020). Indirect biosensor detection strategies for ACA have also been implemented in *E. coli.* First, by pairing the 3-HP responsive regulator, HpdR, from *P. putida* with the biotransformation of ACA to 3-HP via *pa0132* (beta-alanine:pyruvate transaminase) and *ydfG* (NADPH dependent 3-hydroxyacid dehydrogenase). The resulting biosensor demonstrated a ∼10-fold dynamic range in response to exogenous 3-HP (Yuan et al., 2022). In the same study, a whole-cell ACA detection strain of *S. cerevisiae* was employed. Here, a stain of *S. cerevisiae* engineered to convert L-tyrosine to betalamic acid was incubated with exogenous ACA whereby upon import into the cytosol, betalamic acid and ACA react to form β-alanine-betaxanthin, a fluorescent compound (Yuan et al., 2022).

#### 3.5.2 4-Amino butyric acid

Amino butyric acid, or gamma amino butyric acid (GABA), is a monomer for Nylon 4. A biosensor for GABA was developed in zebrafish larvae, based on the responsive periplasmic binding protein Pf622, from a *Pseudomonas fluorescens* strain, which was then used to create a circularly permuted green fluorescent protein (cpGFP) (Marvin et al., 2019). As well, a biosensor for GABA was developed using the regulator and promoter region upstream from the 4-aminobutyrate aminotransferase gene, *gabT,* from *Rhizobium leguminosarum* coupled to *lux-*based reporter biosensor afforded dynamic range of >3.5-fold in the presence of GABA (Pini et al., 2017). Another GABA biosensor was developed in *E. coli* using the GabR regulator from *Bacillus subtilis*, this biosensor afforded a dynamic range ∼140-fold in the presence of GABA (Lebovich and Andrews, 2022). Cyclised GABA, butyrolactam (BL) is also a precursor for Nylon 4, and a lactam biosensor developed in *E. coli* based on the ChnR regulator from *Acinetobacter* sp. was reported to display partial response to BL (∼1.5-fold) (Zhang et al., 2017).

#### 3.5.3 Delta-valerolactam and epsilon-caprolactam

Delta-Valerolactam (DVL) is a monomer for Nylon 5. As well, epsilon-Caprolactam (ECL) is a monomer for Nylon 6 production. A biosensor for DVL and ECL was developed in *E. coli* based on the ChnR regulator from *Acinetobacter* sp. and was reported to display a dynamic range of ∼2.4-fold and ∼2.1-fold in the presence of DVL and ECL, respectively (Zhang et al., 2017). Another biosensor for both lactams was developed in *E. coli* based on the regulator of the nitrile degradation operon, NitR, from *A. faecalis* (Yeom et al., 2018). Through iterative rounds of genetic and protein engineering the optimised NitR-based biosensor, CL-GESS_NitR-L117F_ was reported to afford a dynamic range of >11-fold and ∼10-fold towards DVL and ECL, respectively. Finally, a biosensor was developed in *E. coli* based on the AraC-type regulator, OplR, from the lactam catabolic operon in *P. putida* (Thompson et al., 2020). The optimised OplR-based biosensor, pLACSENS4, was reported to afford a dynamic range of >250-fold in the presence of DVL. While the variant biosensor, pLACSENS3, wherein *OplR* is expressed from a different promoter, demonstrated significant induction in the presence of ECL.

### 3.6 Other monomers and precursors

#### 3.6.1 Acrylic acid

Acrylic acid (ALA) is used in the production of polyacrylic acid (PALA) and as a co-polymer in polyacrylamides. A biosensor for ALA was constructed in *E. coli* based on the Tet-family regulator of the dimethylsulfoniopropionate catabolic operon, AcuR, from *Cereibacter sphaeroides* (formerly *R. sphaeroides*) (Rogers and Church, 2016; Rogers et al., 2015). The AcuR-based biosensor was reported to afford a dynamic range of >90-fold in the presence of ALA. Subsequently, a whole cell biosensor was developed in *E. coli* using an endogenous promoter that was identified as being responsive to ALA. Here, *eGFP* was placed downstream from a copy of the promoter controlling the stress responsive gene, yhcN, to afford a dynamic range of ∼7-fold in the presence of ALA (Raghavan et al., 2019).

#### 3.6.2 Ethanol

Ethanol (EtOH) is used in the production of polyethylene, via ethylene, and can be used to produce butadiene and therefore polybutadiene rubber. A biosensor designed to sense butanol was developed in *E. coli* based on a transcriptional regulator, BmoR, associated with the n-alkane metabolism in *Pseudomonas butanovora* (Wu et al., 2022). The wild-type BmoR-based biosensor was also reported to display a dynamic range of >100-fold in response to EtOH.

#### 3.6.3 Glycerol

Glycerol (GOH) is a polyol with application in the production of polyurethanes. A glycerol biosensor was developed in *Bacillus subtilis* based on the endogenous glycerol uptake antiterminator protein, GlpP, and the corresponding regulated promoter for glpD (Han et al., 2022), the optimised GlpP-based biosensor afforded a dynamic range of >30-fold in the presence of GOH.

#### 3.6.4 Styrene

Styrene (STY) is the monomer of polystyrene. A whole-cell STY biosensor was developed in *Pseudomonas* sp. based on the two-component regulation system from the endogenous styrene catabolic operon involving the sensor StyS and regulator StyR. Here, the *lacZ* reporter gene was inserted downstream from a copy the responsive promoter P*styA* (Alonso et al., 2003). The StyS/StyR-based biosensor afforded a dynamic range of 7-fold in the presence of styrene.

#### 3.6.5 Cyclohexanone

Cyclohexanone (CH) is a precursor for caprolactone which is used in the production of Nylon 6 and Nylon 6,6. A CH biosensor was developed in *E. coli* based on the regulator-promoter pair of the cyclohexanol oxidation operon from A*cinetobacter* sp., ChnR (Steigedal and Valla, 2008). The ChnR-based biosensor afforded a dynamic range of >40-fold in the presence of CH (Kim et al., 2018). Another biosensor for CH was developed in *E. coli* based on the regulator of nitrile degradation operon, NitR, from *A. faecalis* (Yeom et al., 2018). Through iterative rounds of genetic and protein engineering the optimised NitR-based biosensor, CL-GESS_NitR-L117F_ was reported to afford a dynamic range of ∼10-fold in the presence of CH.

#### 3.6.6 Isoprene

Isoprene (IP) is the monomer of synthetic rubber. A biosensor for IP has been reported in *E. coli* and *Pseudomonas putida* based on the transcriptional regulator of the toluene–benzene utilization pathway, TbuT, from *Ralstonia pickettii* (Kim et al., 2018). The TbuT-based biosensor afforded a ∼25-fold dynamic range in both *E. coli* and *P. putida* hosts. Another IP biosensor was developed based on a chimeric transcription factor, AcIA, comprising the AraC transcription factor and IsoA the α subunit of the isoprene monooxygenase from *Rhodococcus* sp. AD45 (Bhat et al., 2023). The optimised AclA-based biosensor afforded a dynamic range of ∼20-fold in the presence of IP.

### 3.7 Biosensor performance and application considerations

**Fig. 2:**
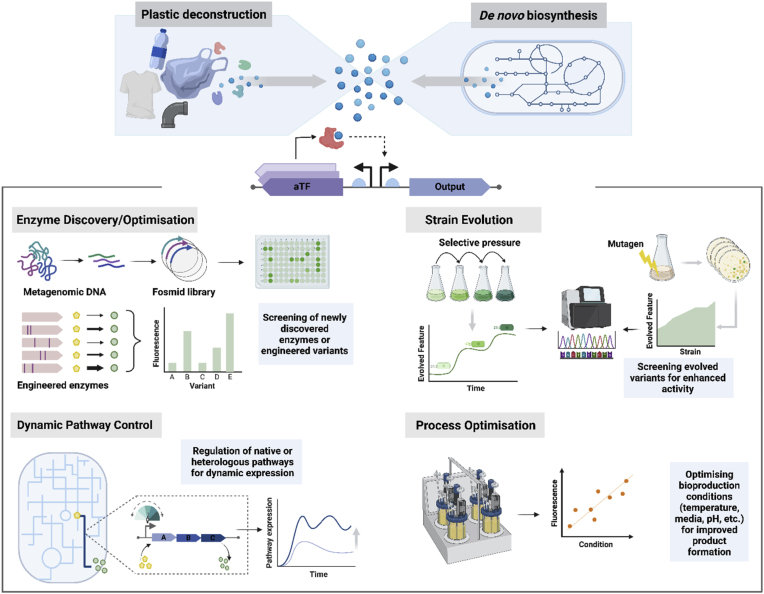
Plastic monomer responsive biosensors have been employed to enhance bio-based plastic deconstruction and monomer production. Biosensor-facilitated metagenomic enzyme screening and enzyme engineering has been applied to both the discovery and improvement of plastic-acting enzymes, as well as over overcoming enzymatic bottlenecks in the *de novo* synthesis of plastic monomers. Further, these plastic biosensors have been used to drive the evolution of high titre bioproduction strains, dynamically regulate *in vivo* pathway expression, and guide process development.

The desired characteristics of a biosensor—such as its response type, sensitivity, specificity, and threshold—are highly dependent on the intended application (Fig. 1, Fig. 2) (Mitchler et al., 2021). For example, for those applications requiring fine-tuned metabolic control or high-resolution product quantification, a linear response may be preferred, while in contrast, a digital (on/off) response may be more desirable for applications requiring a decisive trigger point, such as the switch-like control of metabolic pathways or the binary (pass/fail) screening of enzyme libraries (Alvarez Gonzalez et al., 2024). Likewise, the ligand sensitivity of a biosensor can be engineered based on need; biosensors with high ligand sensitivity can be used for early detection of low metabolite concentrations in processes requiring rapid response or high-resolution differentiation between enzyme variants, while less sensitive biosensors are ideal for detecting only high concentrations and can be used to screen strain/enzyme libraries for high titre product formation. The most universally desirable biosensor attribute is robustness, as it ensures consistent and reliable performance under varying conditions, including fluctuations in environmental factors and process scales. Ultimately, biosensor selection and engineering must align with the specific operational demands of the task, emphasizing the importance of customizing biosensor design to its application for optimal performance (Yu et al., 2023).

## 4 Application of biosensors towards plastic (de)construction

With the linear production and consumption of plastics being increasingly untenable, it is essential that the plastic economy be redesigned. Two key targets for circularising this industry are: enhancing the uptake of renewable plastics and improving plastic recycling. Further, these targets should be addressed synergistically to maximize carbon recirculation and the reduction of GHG emissions (Vidal et al., 2024). The biological production and deconstruction of plastics using enzyme-based biocatalysts and whole-cell systems is a promising approach for achieving improved plastic circularity. The last few decades has seen a surge in the pursuit of biotechnological solutions to the plastics problem, though for the most part, these technologies are in their infancy. Here, we discuss the current and prospective application of biosensors for the advancement of bio-based plastic construction and deconstruction.

### 4.1 Deconstruction

Research aimed at the biocatalytic degradation of commodity plastics has boomed recently, led by the expectation that these technologies will transform the landscape of commercial plastic recycling by permitting more energy efficient processes and improved retention of material value. Currently, however, the number of plastics that can be enzymatically depolymerised is limited to those with relatively easy to hydrolyse chemical linkages, such as esters and amides (PET, PLA, PA, PU), and this biocatalytic depolymerisation is generally slow (Tournier et al., 2023). Expanding and improving this technology will require mining nature for novel enzymes, and further improving the performance of known plastic degrading enzymes to enhance catalytic activity, specificity, thermostability, tolerance to high substrate loadings via directed evolution and structure-based engineering. This work will require the evaluation of large enzyme libraries, a process that can be bottlenecked by low throughput screening techniques when there is no distinct phenotype (ex. cell viability, product fluorescence) (Longwell et al., 2017). Here, genetically encoded biosensors can be employed as an optically detectable proxy for enzyme activity, providing a powerful tool that is conducive to high throughput screening (Fig. 2).

PET is an ester-based plastic used extensively for the manufacture of textiles (polyester fibres) and single-use packaging. As such it has now come to accounting for 12% (by weight) of total global solid waste (Raheem et al., 2019). More than 90 enzymes from Bacteria and Fungi have been described as having PET-degrading activity, reducing this polymer to its ethylene glycol (EG) and terephthalic acid (TPA) monomers. Ongoing work towards improving the catalytic turnover rate/environmental optima of these enzymes is promising. Here, TPA responsive biosensors can be employed as a useful enzyme screening tool. Dierkes et al. (2023), illustrated this when they applied a TPA responsive TphR biosensor with nanomolar range sensitivity from *C. thiooxidans* S23 to detect TPA released from PET plastic by the PET-acting enzymes: *Is*PETase, LCC, and PET40. A fluorescent read-out by the *C. thiooxidans* TPA reporter strain, incubated with the enzyme assay hydrolysates, was detectable within 1.5–2.5 h, a positive trait for high throughput screening. Likewise, design of experiments (DoE) guided engineering was used to create two TPA responsive TphR biosensors with custom performance (VEC1 = a sensitive digital response and DR1 = a linear dose response slope) in *P. putida* (Alvarez Gonzalez et al., 2024). The application of these biosensors was then illustrated towards (i) the sensitive detection of TPA, performed by screening a number of broad-spectrum esterases and lipases against PET plastic using the VEC design, and (ii) quantification of TPA, which was done by comparing LCC-ICCG activity towards PET under variable temperature and pH conditions using the VDR design. The latter biosensor demonstrated utility for semi-quantitative assessment of enzyme variants or critical enzyme parameters. Another interesting approach to TPA detection involved coupling a carboxylic acid reductase (CAR) and luciferase (LuxAB) in *E. coli* to subsequently reduce and oxidise TPA, emitting visible light in the process. This enzyme-coupled bioluminescence biosensor was then used to detect TPA released from PET hydrolysed by LCC, LCC-ICCG, and PES1 enzymes in a semi-quantitative manner (Bayer et al., 2022).

Another class of ester-based plastics are phthalate esters (PAEs), which are used as plasticizers (accounting for 70% of this market) and as additives in paints, adhesives, cosmetics, etc. PAEs can be enzymatically hydrolysed by esterases in a stepwise fashion, initially producing a monoalkyl phthalate followed by free phthalate. The recently characterised enzymes *Go*Est15 and *Go*ESTM1 from *Gordonia* sp. are capable of hydrolysing a broad range of PAEs, with *Go*EST15 (responsible for the first hydrolysis step) being the rate-limiting step (Huang et al., 2019). Li et al. (2022) performed biosensor mediated directed evolution of *Go*EST15 to improve its activity using a phthalate responsive XylS biosensor. A *Go*EST15 library was created using saturation mutagenesis of the active site, and then co-transformed into *E. coli* with *Go*ESTM1 and a PA responsive biosensor (XylS-K38R-L224Q). This library was screened against dibutyl phthalate (DPB) and ranked for fluorescence. One of the positive hits, *Go*EST15-V3, was found to have 2-fold improved activity towards DBP than the wildtype enzyme *in vitro*, directly evidencing the value of biosensors for protein engineering.

Genetically encoded biosensors have also been applied to the discovery of putative plastic degrading enzymes. Uchiyama and Miyazaki (2010) recently screened a metagenomic library obtained from activated sludge for amidase activity using a benzoate responsive biosensor assay termed PIGEX (product-induced gene expression). Amidases catalyse the hydrolysis of an amide, leading to the formation of a carboxylic acid. Polyamides (PAs), also known as nylons, are thermoplastics used extensively for textiles. Here, *E. coli* cells harbouring a metagenomic fosmid library were individually co-cultured with the PIGEX *E. coli* reporter strain in the presence of benzamide. From this, three novel amidases were identified, and found to have activity not only towards benzamide but also towards a number of amino acid amides, including glycine amide, a constituent of nylon 2,6. In another example of amidase screening, Raghavan et al. (2019) developed an acrylic acid (ALA) sensing strain of *E. coli* by pairing the native ALA responsive promoter P_yhcN_ with GFP expression, and then used it to screen a mutant library of the *G. pallidus* RAPc8 amidase for improved amide hydrolysis of acrylamide. Here, four variants demonstrated improved activity compared the wild type, with variant RAPc8-C60 (M45L; A77T; M203V; D294N; K342E) having a 1.6-fold improvement in *k*_*cat*_/*K*_*M*_. While these amidases were not examined for PA depolymerisation, their discovery broadens the pool of potential PA-acting enzymes, of which there are relatively few candidates.

Biocatalytic degradation of plastics with carbon-carbons bonds (such as PE, PS, PP, and PVC) is considerably more challenging than the ester and amide-based plastics. While a large number of organisms (both prokaryotes and eukaryotes) have been affiliated with the degradation of these plastics, the enzymes and mechanisms behind these activities are not fully understood (Danso et al., 2019; Tournier et al., 2023). Here again, biosensors could be used to mediate discovery. For example, styrene and acrylic acid responsive biosensors (Alonso et al., 2003; Rogers and Church, 2016) could be used to mine enzymes capable of polystyrene (PS, used extensively for packaging and insulation) and polyacrylic acid (PALA, used commonly for water absorption in diapers) depolymerisation. Preliminarily, Puhakka et al. (2022) demonstrated that an ALA responsive-luciferase reporter system in *E. coli* could detect ALA monomers released from PALA subjected to mechanical stress, implying this system could find use for screening enzyme-mediated depolymerisation as well.

Limitations exist, however, in the application of biosensors for evaluating plastic deconstruction. Most biosensors display high substrate specificity, requiring monomerization of the plastic substrate to their cognate ligand for recognition. For example, the TPA responsive biosensors, built with components from *Zhizhongheella caldifontis* and *Pigmetiphaga litoralis*, do not respond to the hydrolysis intermediates, methyl-(2-hydroxyethyl) terepthalate (MHET) or bis-hydroxyethyl terepthalate (BHET), released in addition to TPA by PET-acting enzymes (Alvarez Gonzalez et al., 2024). Thus, relying on a strict TPA responsive biosensor to characterise novel or engineered PETases may not capture the full landscape of catalytic activity. As such, this restricts the application of biosensors for the evaluation of enzymes not directly responsible for monomerization. Another example of limitations are those enzymes involved in the surface modification of recalcitrant plastics. The biocatalytic introduction of functional groups – hydroxyl, carbonyl, and esters – to the backbone of carbon-carbon based plastics, such as PE, via free radical and redox reactions is an important first step to degradation (Jin et al., 2023); and is one that cannot be directly coupled to a biosensor output. Instead, here, the development of enzymatic cascades that result in detectable monomers could be implemented to assess the early modification of plastics.

### 4.2 Construction

Numerous commodity plastics can now be produced from renewable feedstocks, including bio-PET, -PE, -PP, -PS and -PVC. Many of the monomers that constitute plastics can be made directly via microbial biosynthesis from biomass derived compounds (carbohydrates, lipids, etc), or by the successive chemical conversion of microbially synthesised precursors. These monomers then undergo conventional chemical polymerisation to make drop-in bioplastics or novel polymers (Rosenboom et al., 2022). Notably, the term bioplastic encompasses both those made entirely or partially from renewably source monomers, the latter also being termed hybrid bioplastic. Choi et al. (2023) offers an excellent review on the current landscape of biological plastic production and its commercialisation.

In 2023 approximately 2.2 Mt of biobased plastics were produced globally, a number that is forecast to increase to 7.4 Mt by 2028 (Europeanbioplastics, 2023). Despite these strides, this still represents a small fraction of the 350–400 Mt of total plastic currently being generated annually (Geyer et al., 2017). Improving commercial bioplastics production begins with microbial strain development, pushing microbial cell factories towards efficient, high titre production. In the last few decades, tools and techniques from the fields of metabolic engineering, synthetic biology and microbial adaptive evolution have been leveraged for high throughput genetic diversification using enzyme-genome-, pathway-, and metabolic flux-engineering (Han et al., 2023). Our capacity to generate large genetic libraries, however, has outstripped our ability to screen them. The bottleneck of screening these microbial strain libraries for high titre production can be addressed by using biosensors, with numerous studies illustrating their application for improving plastic monomer synthesis (Fig. 2).

#### 4.2.1 Screening of enzyme libraries and overcoming enzymatic bottlenecks

A common bottleneck to high titre microbial production is low/inefficient enzyme activity. Strategies for overcoming this typically involve screening enzyme homologues or engineered variants for improved catalytic efficiency. This has been the case for glucaric acid (GCA), a versatile platform chemical that can be used in the production of nylon and other plastics. High titre microbial production of GCA is limited by the activity of the myo-inositol oxygenase (MIOX) enzyme, which converts myo-inositol to glucuronic acid (GRA) before its further conversion to GCA by a glucuronate dehydrogenase (Udh). In an effort to overcome this bottleneck step, Nash and Prather (2023) successfully utilised a GRA responsive biosensor, ExuR, to screen four MIOX homologues in *E. coli*, successfully demonstrating the biosensor could be applied to identify the homologue with best conversion of exogenously supplemented myo-inositol (the MIOX from *Flavobacterium johnsoniae*). In another study, MIOX homologues were once again screened in *E. coli* using a GCA responsive CdaR biosensor (Rogers et al., 2015). Here, four MIOX orthologues were paired with a constitutively expressed Udh and co-transformed with the CdaR biosensor to monitor GCA production *in situ*. It was found that the four MIOX strains produced fluorescent signals across a 20-fold range, correlating well with GCA titres as measured by HPLC, and identifying the MIOX from *Mus musculus* as the superior candidate. Another study used the GCA responsive CdaR biosensor to screen a mutant library of small ubiquitin-like modifier (SUMO)-MIOX variants in *E. coli* via sequential production and biosensing cultivations. Nine variants demonstrated a 15% improvement in fluorescence over the wild type, leading to the identification of the highly active SUMO-MIOX-D82Y-S173N mutant, which was capable of over a two-fold improvement in GCA titres compared to the wild type sequence from exogenously supplied myo-inositol (Zheng et al., 2018).

Some lactams have also been targets for microbial synthesis, being important building blocks for the production of nylons. *In vivo* production of Epsilon-caprolactam (ECL) relies on the cyclisation of 6-aminocaproic acid (6-ACA) by an acyl-CoA ligases (cyclase). Thompson et al. (2020) used an ECL responsive OlpR biosensor to screen five cyclases in *P. putida* for their ability to produce ECL from exogenous 6-ACA, using a dual plasmid system. Another example of cyclase screening involved the use of an engineered ECL responsive NitR biosensor (CL-GESS_NitR-L117F_) to screen a marine metagenomic library for novel enzymes (Yeom et al., 2018). Here, the metagenomic fosmid library was co-transformed with CL-GESS_NitR-L117F_ to screen for cyclisation of exogenously supplied 6-ACA in *E. coli*, and successfully identified a novel 3-hydroxybutyrate dehydrogenase (CF3HBD) from *Citrobacter freundii* with promiscuous cyclisation activity. Further, the CF3HBD was then paired with CL-GESS_NitR-L117F_ to screen for the presence of 5-AVA (5-aminovaleric acid) and 6-ACA ω-amino fatty acids, demonstrating the utility of an enzyme coupled biosensor for detection of linear precursors. To overcome the rate limiting cyclisation step of 5-AVA to delta-valerolactam (DVL), Zhao et al. (2023b) used an engineered DVL responsive ChnR-B1/Pb-E1 biosensor to evaluate the benefit of dynamically regulating the expression of three different terminal cyclases (*act, orf26, and caiC*) for *de novo* DVL production by *C. glutamicum* from L-lysine. In all cases, this biosensor mediated dynamic expression resulted in improved product titres compared to constitutive expression, with the strain harbouring the β-alanine CoA transferase (*act*) cyclase ultimately achieving 12.3 g/L DVL in fed batch bioreactor.

Microbial production of diamines, such as putrescine (PUT), cadaverine (CAD) and 1,6-hexanediamine, has been an active area of research due to their application in the production of PA plastics. Putrescine can be synthesised from L-arginine, making *C. glutamicum* – an industrial amino acid production host – an attractive chassis for its production. However, *C. glutamicum* cannot natively synthesise PUT, instead it requires heterologous expression of an ornithine decarboxylase (ODC) to produce this compound. Zhao et al. (2021) co-transformed an engineered PUT responsive CmgR biosensor (pSenPutI_152T_) with four different ODC enzymes to evaluate *in situ* PUT production. For all candidates, fluorescence signal correlated well with PUT quantification via HPLC. The ODC argB H268E was identified as the best candidate, resulting in over 50 mM of product after a 72-h cultivation.

3-hydroxyproprionate (3-HP) is a versatile platform chemical that can be converted into other plastic monomers or can itself be polymerised into homopolymer or copolymer polyhydroxyalkanoates (PHAs). 3-HP can be produced from glycerol via a glycerol dehydratase (GDHt) and aldehyde dehydrogenase (ALDH), however low activity from the ALDH has been found to result in cell inhibition due to the accumulation of the toxic 3-hydroxypropionaldehyde (3-HPA) intermediate – negatively impacting 3-HP titres. Seok et al. (2018) sought to overcome this bottleneck by constructing and screening a KGSADH (alpha-ketoglucaric semialdehyde dehydrogenase) enzyme library whereby the aldehyde-binding site was targeted for diversification. This library was co-expressed in *E. coli* with a 3-HP responsive MmsR biosensor controlling expression of *tetA*, a tetracycline/H+ antiporter that confers resistance to this antibiotic. Following selective cultivation, a KGSADH variant containing four-point mutations was found to be capable of producing 25% more 3-HP than the wild-type sequence (5.08 g/L versus 4.07 g/L). Further, the KGSADH variant identified in this study was compared to one previously isolated from the same library via a low-throughput NADH absorbance screen (Park et al., 2017), and found to be superior. This illustrates the strength of high-throughput biosensor screens for identification of variant enzymes with desired properties.

Muconic acid (MA) is a dicarboxylic acid with enormous potential for the synthesis of multiple plastic polymers, including nylons, polyesters, and PET, making it a common target for microbial production. It can be made by diverting 3-hydroshikimate (DHS) from central metabolism through to protocatechuic acid (PCA), catechol and finally, MA. In *S. cerevisiae*, this requires heterologous expression of *aroZ* (dehydroshikimate DHS dehydratase), *aroY-BCD* (PCA decarboxylase), and *catA* (catechol 1,2 dioxygenase). The decarboxylation step of PCA to catechol has been recognised as rate limiting, leading Skjoedt et al. (2016) to introduce single or multiple copies of different PCA decarboxylase subunits from *Klebsiella pneumonia* (*aroY* subunits B and C) into the genome of *S. cerevisiae* in an attempt to alleviate this bottleneck. A six-strain library was then screened post fermentation for MA production using an engineered MA responsive BenM biosensor, and it was found that the best performer carried multiple copies of PCA decarboxylase subunits B and C, as well as an additional copy of the endogenous transketolase (*tkl1*), resulting in 1.39 mM of MA. To further de-bottleneck this PCA decarboxylation step, Jensen et al. (2021) coupled OrthoRep mediated continuous *in vivo* mutagenesis of the B and C *iso* subunits of *aroY* decarboxylase with *in situ* selection for high MA production using the BenM based biosensor. After iterative rounds of evolution and selection, a variant of the AroY subunit B (AroY. B_P146T) was identified as being capable of a 13.7-fold higher MA titre compared to the wild-type protein when expressed in the same genetic background.

#### 4.2.2 Broad screening of genetic libraries

Engineering high titre microbial production can be challenged by the complexity of the structure and regulation of metabolic networks, often leading to non-intuitive and non-linear effects. More holistic approaches, such as directed/adaptive evolution or the generation of random/rational genetic libraries, have afforded impressive improvements in target molecule biosynthesis (Cobb et al., 2013). In the case of MA production, another strategy employed to overcome the rate limiting PCA decarboxylation step in *S. cerevisiae* involved creating a semi random multi-copy integration library of *aroY*-B and *aroY*-C (iso) in a strain genomically harbouring the rest of the MA biosynthetic pathway and a MA responsive BenM biosensor controlling expression of *kanMX*, the gene that confers weak resistance to the antibiotic G418 (Snoek et al., 2018). This library was then cultured in the presence of G418 to confer selection pressure towards high titre MA producers. One of the isolates from this evolved population was capable of producing 2 g/L MA and was found to possess seven copies of *AroY*-B and *AroY*-Ciso in its genome. Leavitt et al. (2017) used a similar strategy to improve flux through the shikimate pathway for improved DHS precursor availability for MA production. Here, they generated *S. cerevisiae* libraries using ethyl methanesulfonate-induced mutagenesis and evolved them towards high aromatic amino acid (AAA) production by pairing an AAA responsive Aro9p biosensor with expression of the G418 resistance gene, selecting for over producers by sequentially increasing the concentration of G418 and the anti-metabolite 4-FP (4-fluorophenylalanine). High producers were then isolated and transformed with the MA biosynthetic pathway, with each achieving approximately a threefold improvement in the titre of MA and its pathway intermediates compared to the parental strain.

Another study created a library of *S. cerevisiae* harbouring the MA production pathway and an engineered MA responsive BenM biosensor (MP02_D04) using UV mutagenesis. The library was sorted for high and low fluorescence output, with one isolate, Mut131, identified as capable of 49.7% more MA production than the parental strain (Wang et al., 2020). Mut131 was found to have number of missense mutations in native genes as well as duplications of the MA pathway genes, *aroZ* and *catA*. Others have integrated the MA biosynthesis pathway into knockout (KO) and overexpression (OEx) libraries of *S. cerevisiae* using CRI-SPA screened the impact of these individual gene modifications on product formation using the BenM biosensor, identifying a number of novel targets that impact product formation (Cachera et al., 2024). While another interesting study screened a library of MA producing *P. putida* Δ*gcd* (glucose dehydrogenase) that had been adaptively evolved for improved growth on glucose. Here, an engineered MA responsive CatM biosensor (pCatM_C2) was used to identify isolates with both improved growth and MA synthesis (Bentley et al., 2020).

To improve heterologous 3-HP production by *E. coli*, Seok et al. (2021) paired 3-HP synthesis with cell survival in the presence of the antibiotic tetracycline by placing *tetA* expression under the control of a 3-HP responsive MmsR biosensor. After several rounds of selective evolution, two isolates capable of 37% and 48% more 3-HP synthesis than parental strain were achieved. It was found that they, respectively, possessed mutations in *cynA*, encoding an adenylyl cyclase, and CRP, encoding the cAMP receptor protein – both of which influence transcription of genes associated with carbon metabolism. Combining both mutations in the presence of the 3-HP production pathway resulted in 55 g/L of product.

Another target for microbial production is the non-proteinogenic amino acid, 3-aminopropionic acid (ACA), which has a number of commercial applications including as a constituent of plastics. ACA can be biosynthesized from the TCA cycle intermediate, fumarate, via the activity of an aspartase (*aspA*) and an L-aspartate-α-decarboxylase (*panD*). To evolve an ACA producing strain of *E. coli* to achieve higher titres, Yuan et al. (2022) used genome-wide Tn10 transposon insertional mutagenesis to diversify the population. To select for high producers, this library was then transformed with a 3-HP responsive HpdR biosensor as well as the requisite heterologous genes (*pa0132* and *ydfG*) to convert ACA to the proxy molecule, 3-HP. From this, ribonuclease E (*rneE*) was identified as a novel target that influences ACA product titres, with a *rneE* knockdown strain achieving 17% more ACA than the parental strain.

#### 4.2.3 Fine-tuning pathway regulation for expression

Metabolic pathways/networks have evolved to finely balance the generation and consumption of building block and energy; thus, rewiring these pathways requires the same consideration. Here, dynamic gene/pathway regulation can be employed to mimic native metabolism (Fig. 2). Itaconic acid (IA) is a platform chemical with broad application for plastic polymer synthesis. Microbially, it can be derived from the TCA cycle intermediate, *cis*-aconitate, by the action of a *cis*-aconitate decarboxylase (CadA), however over-expression of *cadA* can impair culture growth, likely due to associated metabolic burden (Hanko et al., 2018). To identify optimal *cadA* expression in *E. coli*, one group designed a single plasmid relay system whereby *cadA* expression was governed by an arabinose inducible expression system to produce IA, which would then elicit a fluorescent signal from an IA inducible ItcR biosensor. From this, a titration of the arabinose inducer could be evaluated to identify the optimal concentration for *cadA* expression for IA synthesis (Hanko et al., 2018). In another elegant example, a sensor-regulator system based on the MA responsive CatR biosensor was developed to simultaneously upregulate precursor production and downregulate competing carbon flux in response to the *in situ* MA levels produced by *E. coli* harbouring a biosynthetic pathway to produce MA from chorismate via salicylic acid (SCA) (Yang et al., 2018). The genes targeted for dynamic upregulation were *entC* (isochorismate synthase) and *pchB* (isochorismate pyruvate lyase), to provide the SCA precursor, while the gene targeted for RNAi mediated dynamic downregulation was *ppc* (phosphoenolpyruvate carboxylase), which is involved in incorporating PEP into the TCA cycle. This bifunctional dynamic control strain demonstrated improved product formation compared to constitutive expression, achieving 1.86 g/L of MA after 48 h (compared to 316.2 mg/L).

Another example of metabolic engineering where balanced pathway expression was considered is for the hydroxy monocarboxylic acid, glycolic acid (GA), which can be polymerised to make the biodegradable bioplastic, (poly)glycolic acid (PGA). GA can be synthesised microbially from the glyoxylate shunt of the TCA cycle via the activity of a glyoxylate reductase (YcdW), however balancing flux distribution between glycolate synthesis and the TCA cycle is imperative to high titre production. Xu et al. (2021) created an *E. coli* library whereby 22 gradient strength promoters and 5′-untranslated regions (PUTRs) were randomly inserted upstream of the *ycdW* (glyoxylate reductase), *aceA* (isocitrate lyase), and *gltA* (citrate synthase) genes involved in GA synthesis to create a plasmid-based expression library. This library was then screened for high titre GA synthesis by pairing production with survival in the presence of tetracycline via a co-expressed GA responsive GlcC biosensor that controlled expression of the tetracycline resistance gene, *tetA*. Evaluation of 20 of the highest producers revealed that medium/high expression of *ycdW* and *gltA* and low expression of *aceA* was the best expression stoichiometry for GA production.

#### 4.2.4 Process optimisation

An integral aspect of high titre bio-production is robust process development. Here, optimizing process variables such as: media composition, seeding conditions, carbon source, culturing conditions, etc, and overcoming population heterogeneity are integral aspects. Thus, the utilization of rapid high throughput screening platforms, such as biosensors, for process optimisation would be a considerable asset (Fig. 2). Rogers and Church (2016) demonstrated this when they used a proxy molecule, acrylic acid (ALA), and an ALA responsive acuR biosensor to determine the optimal concentrations of cerulenin and IPTG for 3-hydroxyproprionate (3-HP) production from glucose by *E. coli*. Here, cerulenin inhibited fatty acid synthesis, enriching the intracellular abundance of malonyl-CoA, while IPTG induced expression of *mcr* (bifunctional malonyl-CoA reductase) to shunt malonyl-CoA towards 3-HP synthesis. Under optimised process parameters, this strain produced approximately 2.8-fold more 3-HP related fluorescence and resulted in 4.2 g/L 3-HP after fermentation. In another example, the impact of seeding culture density and glucose/yeast extract addition were evaluated to identify optimal conditions for β-alanine production by engineered *E. coli* (Yuan et al., 2022). Product quantification was performed by incubating the post-fermentation supernatants with a β-alanine-betaxanthin producing yeast biosensor strain. Further, another interesting demonstration of the utility of biosensors for fermentation optimisation involved the screening of genetic heterogeneity in a population after a fermentation optimisation step in order to identify individual high performers. Zhi et al. (2023) used an adipic acid (AA) responsive BenM biosensor to screen an AA producing *E. coli* population for high-performers after individual fermentation optimisation experiments for media composition, carbon source, and pathway precursor supplementation. From this, an individual capable of 18.8-fold higher AA production than the initial strain was identified.

Ultimately, as microbial strain development becomes a more integrated process, exploiting both rational design and evolution guided-engineering strategies to explore vast genetic sequence space, biosensor-mediated chemical detection and quantification has emerged as a critical aid to guide high titre bio-production. Further, the implementation of biosensors as a tool to fine-tune metabolic flux through the dynamic regulation of native and engineered pathways has, and will continue, to make impressive strides. Recently, a multi-layered dynamic regulatory system was constructed to improve GCA production in *E. coli* by independently modulating two points of metabolism (Doong et al., 2018). Here, a quorum sensing biosensor was used to separate growth and product formation by autonomously downregulating the glycolytic enzyme Pfk-1 (instead redirect carbon to GCA synthesis) and a *myo*-inositol responsive IspA biosensor was used to regulate expression of the rate limiting MIOX enzyme. Application of the dual system resulted in a 4-fold improvement in product formation. Ultimately, as our understanding of microbial metabolism and the library of available biosensors grows, so too will the complexity of artificial genetic circuits we can build.

## 5 Conclusion

The proven value of genetically encoded biosensors in advancing the biological construction and degradation of plastics calls for an expanded repertoire of available sensors. For decades, biologists have observed the evolution of microbial communities as they adapt to remediate xenobiotics in contaminated environments. A crucial aspect of these novel metabolic pathways is the development of appropriate regulatory mechanisms (Copley, 2000). For example, soils contaminated with non-native compounds such as polychlorinated biphenyls (PCBs), previously used as coolants and plasticizers, or pentachlorophenol (PCP), previously used as a pesticide, have led to the emergence of novel catabolic pathways that are transcriptionally regulated by either the starting xenobiotic or a pathway intermediate (Flood Jake and Copley Shelley, 2018; Fujihara et al., 2023). Similarly, microbial communities of the plastisphere offer a promising avenue for discovering novel plastic-responsive biosensors (Gambarini et al., 2021; Rillig et al., 2024). A notable discovery is the *tph* operon from Comamonas sp., which encodes a TPA catabolic pathway regulated by the TPA-responsive TphR transcriptional activator (Kasai et al., 2010). A homologous operon was also identified in *Ideonella sakaiensis*, a bacterium isolated from the sediment of a PET bottle recycling site, able to degrade PET and assimilate the breakdown products (Yoshida et al., 2016). Continuing improvements in bioprospecting, sequencing capacity, omics technologies, and computational biology will accelerate discovery of novel plastic responsive transcriptional biosensors from the plastisphere.

Undoubtedly, however, microbial pathways for the remediation of many plastics are still in the early stages of evolution, with the activity and specificity of associated regulators not yet fully fit for purpose. In such cases, synthetic biology can be employed to create novel biosensor variants with desired ligand specificity. Site directed mutagenesis and computationally guided *de novo* design of transcription factor-ligand binding domains has expanded biosensor substrate profiles (De Paepe et al., 2017; Machado et al., 2019; Snoek et al., 2019). A recent example is the work done by Javanpour and Liu (2021) where a muconic acid (MA) responsive BenM biosensor was evolved using the hypermutation system, OrthoRep, towards the non-cognate ligand, adipic acid (AA). While another study computationally redesigned the RbsB ribose binding protein (a periplasmic substrate-binding protein) for novel 1,3-cyclohexanediol and cyclohexanol binding, the latter being a precursor to cyclohexanone (an intermediate in the production of nylon and PCL). *In vivo* library screening that paired ligand binding to a signal cascade terminating in *gfp* expression validated the successful alteration of substrate specificity, and the development of a novel periplasmic binding protein (PBP) based biosensor (Tavares et al., 2019). An ongoing engineering challenge, however, is being able to control the sensor-ligand specificity, as most engineering efforts result in a biosensor with broadened promiscuity as opposed to an absolute shift in ligand recognition (De Paepe et al., 2019). Xi et al. (2023) offers an excellent overview of recent advances in engineering biosensor-ligand specificity.

In tandem to the development of better plastic production and deconstruction technologies, a paradigm shift in the way plastic is viewed is required. Currently, a culture of convenience has normalised single-use plastics and fostered complacency, ignoring the fact that their end-of-life far exceeds their brief consumer lifespan (Lau et al., 2020). It is imperative to prioritise strategies that emphasize reduction and reuse of plastic. Education and awareness initiatives play a pivotal role in enacting societal shifts towards more sustainable practices, empowering individuals to make informed choices. Simultaneously, comprehensive and forward-thinking policy measures that prioritise reduction, reuse, and innovation are essential for driving systemic change to the plastic economy (World Economic Forum E. M. F. a. M. c., 2016).

## CRediT authorship contribution statement

**Micaela Chacón:** Writing – review & editing, Writing – original draft, Formal analysis. **Neil Dixon:** Writing – review & editing, Writing – original draft, Supervision, Funding acquisition, Formal analysis.

## Declaration of competing interest

The authors declare that they have no known competing financial interests or personal relationships that could have appeared to influence the work reported in this paper.

## Data Availability

No data was used for the research described in the article.
